# Flow-diverter stents combined with flow-T stenting-assisted coiling for the treatment of a large basilar apex aneurysm: a case report with a 9-month follow-up

**DOI:** 10.3389/fneur.2023.1247549

**Published:** 2024-01-19

**Authors:** Hao Wang, Jingyu Zhang, Huajiang Yang, Shi Zeng, Tengyun Guo, Lunshan Xu, Donghong Yang

**Affiliations:** ^1^Department of Neurosurgery, Army Medical Center of PLA, Chongqing, China; ^2^Department of Neurosurgery, People's Hospital of Chongqing Banan District, Chongqing, China; ^3^Department of Neurosurgery, The First Affiliated Hospital of Chongqing Medical University, Chongqing, China

**Keywords:** flow-diverter devices, aneurysm, basilar apex aneurysms, supraclinoid aneurysms, endovascular

## Abstract

**Background:**

Endovascular or surgical treatment of wide-neck, large basilar apex aneurysms is challenging. We present a novel concept for the treatment of complex basilar apex aneurysms using flow-diverter devices combined with the flow-T stenting-assisted coiling technique. Assess the efficacy and safety profile of the technique in this complex aneurysm.

**Case description:**

A patient with multiple unruptured intracranial aneurysms underwent staged treatment. A large basilar apex aneurysm was treated with a flow-diverter stent combined with a flow-T stenting-assisted coiling technique in the first stage, and a giant supraclinoid aneurysm was treated with a flow-diverter stent applied in the second stage. Clinical presentations, technical details, intra- and perioperative complications, and clinical and angiographic outcomes were recorded, with a 9-month follow-up.

**Results:**

The patient achieved full neurologic recovery postoperatively. Cerebral angiography performed postoperatively showed revascularization, good laminar flow, and no in-stent or adjacent stenosis.

**Conclusion:**

Flow-diverter stents combined with flow-T stenting-assisted coiling for the treatment of giant basilar apex aneurysms is a feasible technique with efficacy demonstrated at a 9-month follow-up. Staged endovascular treatment of multiple intracranial aneurysms may be a safe and viable option.

## 1 Introduction

The proportion of basilar apex aneurysms (BAAs) to posterior circulation aneurysms ranges from 20% to 40% (1). Basilar apex aneurysms usually present with rupture, signs, and symptoms of mass effect or thromboembolism or are found incidentally. For complex BAA cases, surgical treatment with bypass surgery and aneurysm clipping was a possible treatment in the past, but it required surgical experience and had a high complication rate (2).

The advent of flow-diverter devices has allowed the treatment of wide-neck, large aneurysms with promising clinical and angiographic outcomes (3–5). However, there are still some limitations when it comes to its use in the management of aneurysms in the posterior circulation. These problems are mostly related to their anatomic location and include the risk of occlusion of the posterior cerebral artery (PCA) and superior cerebellar arteries, ischemic lesions of the brain stem caused by perforator artery coverage, and delayed rupture of the treated aneurysms (6–8).

The purpose of this case study was to report the successful completion of definitive coil embolization of wide-neck, large basilar apex aneurysms using flow-diverter stents combined with the flow-T stenting technique, and the effectiveness of this technique was confirmed by a 9-month follow-up. We describe our experience of the feasibility, safety, and efficacy of the procedure in this specific anatomical condition.

## 2 Case description

**Figure 1:**
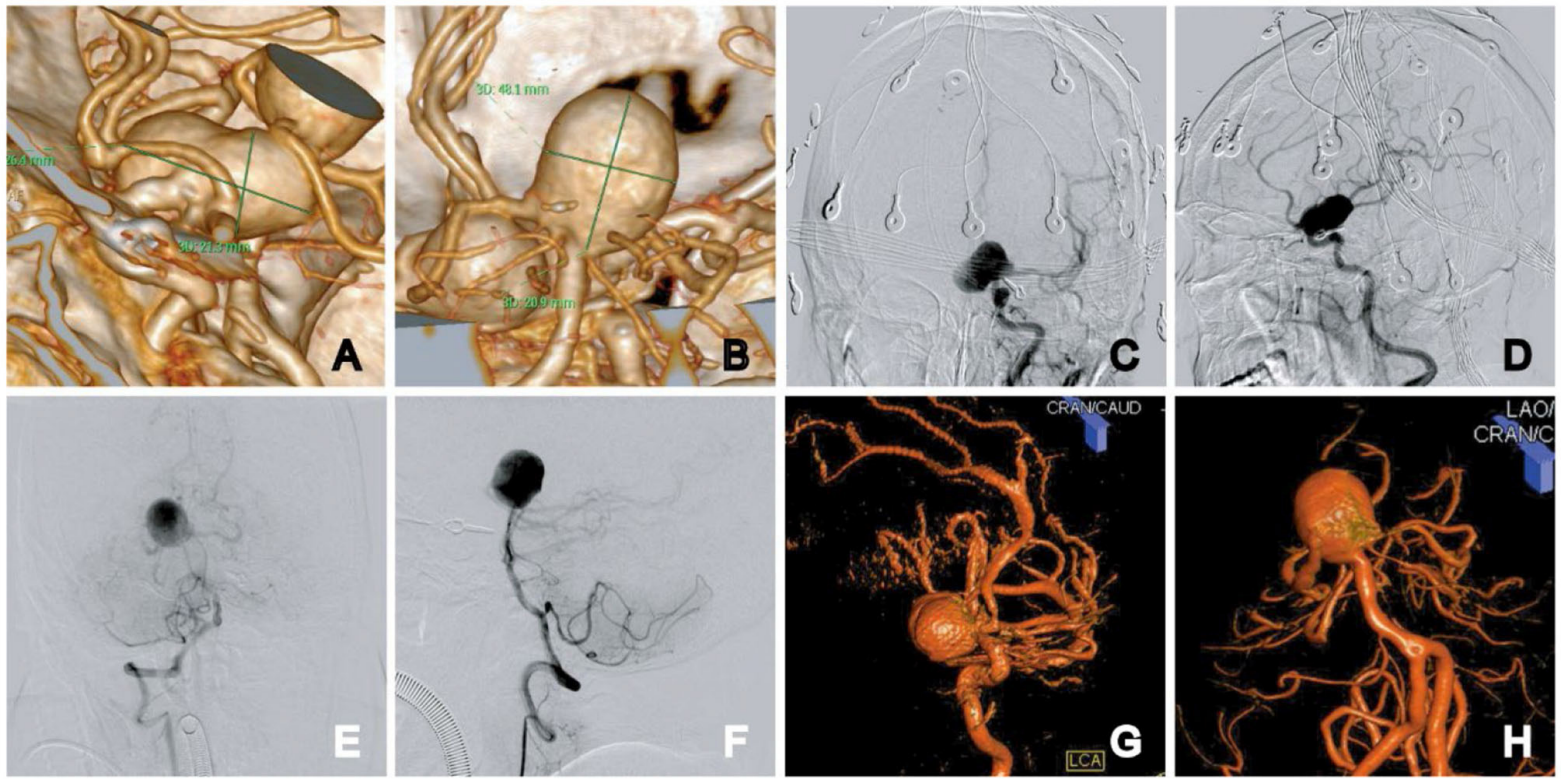
Initial diagnostic workup. CT angiogram images show the left ICA supraclinoid aneurysm and the basilar apex aneurysm: 3D reconstruction measurement, the aneurysm size was 26.4 × 21.3 mm in the left ICA supraclinoid aneurysm **(A)** and 20.9 × 18.1 mm in the basilar apex aneurysm **(B)**. **(C–F)** Anteroposterior and lateral views of the left ICA supraclinoid aneurysm **(C, D)**. Anteroposterior and lateral views of bilateral vertebral arteries **(E, F)**. **(G, H)** 3D rotational angiography of the supraclinoid aneurysm **(G)** and the basilar apex aneurysm **(H)**.

A 65-year-old woman was admitted to the neurosurgery department of our hospital in September 2021 with a headache. No neurological abnormalities were observed. Past medical history: the patient underwent aneurysm clipping in 1998 for a ruptured right posterior communicating aneurysm with subarachnoid hemorrhage, and no other aneurysm was found at that time. However, imaging data were missing. Computed tomography angiography and diagnostic digital subtraction angiography showed a giant supraclinoid aneurysm of the left internal carotid artery (Dmax 26.4 mm × neck 10.0 mm) and a large basilar apex aneurysm (Dmax 20.9 mm × neck 18.1 mm). In the basilar apex aneurysm with quadrifurcation of arteries, both posterior cerebral arteries and superior cerebellar artery emanate from the neck of the basilar apex aneurysm (Figure 1).

**Figure 2:**
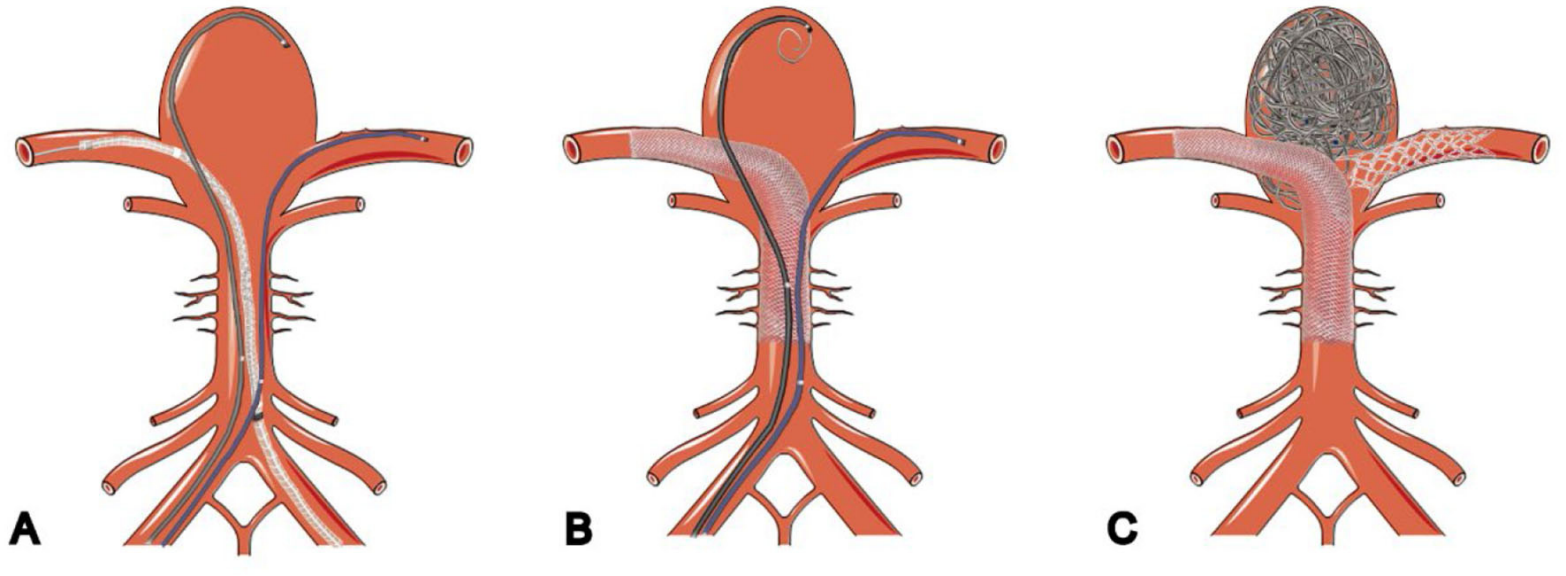
Schematic illustration of flow-diverter stents combined with flow-T stenting-assisted coiling technique. **(A)** Microcatheter deployed into aneurysms and bilateral posterior cerebral arteries. **(B)** Pipeline embolization device deployed into PCA and coils deployed through microcatheters in the aneurysm. **(C)** Stent placed in a T shape after the aneurysm is embolized.

Because the aneurysm was incapable of coiling or neck clipping and given the risks of sacrificing basilar perforators and recurrence, we decided to treat the aneurysm with flow diversion. This technique diverted the flow from the aneurysm, allowing it to thrombose slowly. The aneurysm located at the basilar apex was the initial focus of treatment. The chosen approach used a pipeline embolization device (PED; Medtronic, Irvine, CA, USA) combined with flow-T stent-assisted coil embolization. The technical process is depicted in Figure 2. Treatment of the supraclinoid aneurysm was initiated via PED-assisted coil embolization 2 weeks after the first therapeutic intervention. This is because simultaneous treatment of anterior and posterior circulation aneurysms can be fatal with thrombotic or bleeding complications. Moreover, the patient was an older individual, the physical condition was general, and the operation time was long, which increased the incidence of complications. After discussing the off-label use of the device, the patient agreed to a staged treatment regimen with flow diversion.

The patient was administered dual antiplatelet therapy (100 mg aspirin and 75 mg clopidogrel) 7 days before the procedure. Treatment on thrombosis elastic figure detection arachidonic acid (AA inhibition rate) and adenosine diphosphate receptor pathways induced by blood plate inhibition rate (ADP inhibition rate) met the requirements.

### 2.1 Intervention

**Figure 3:**
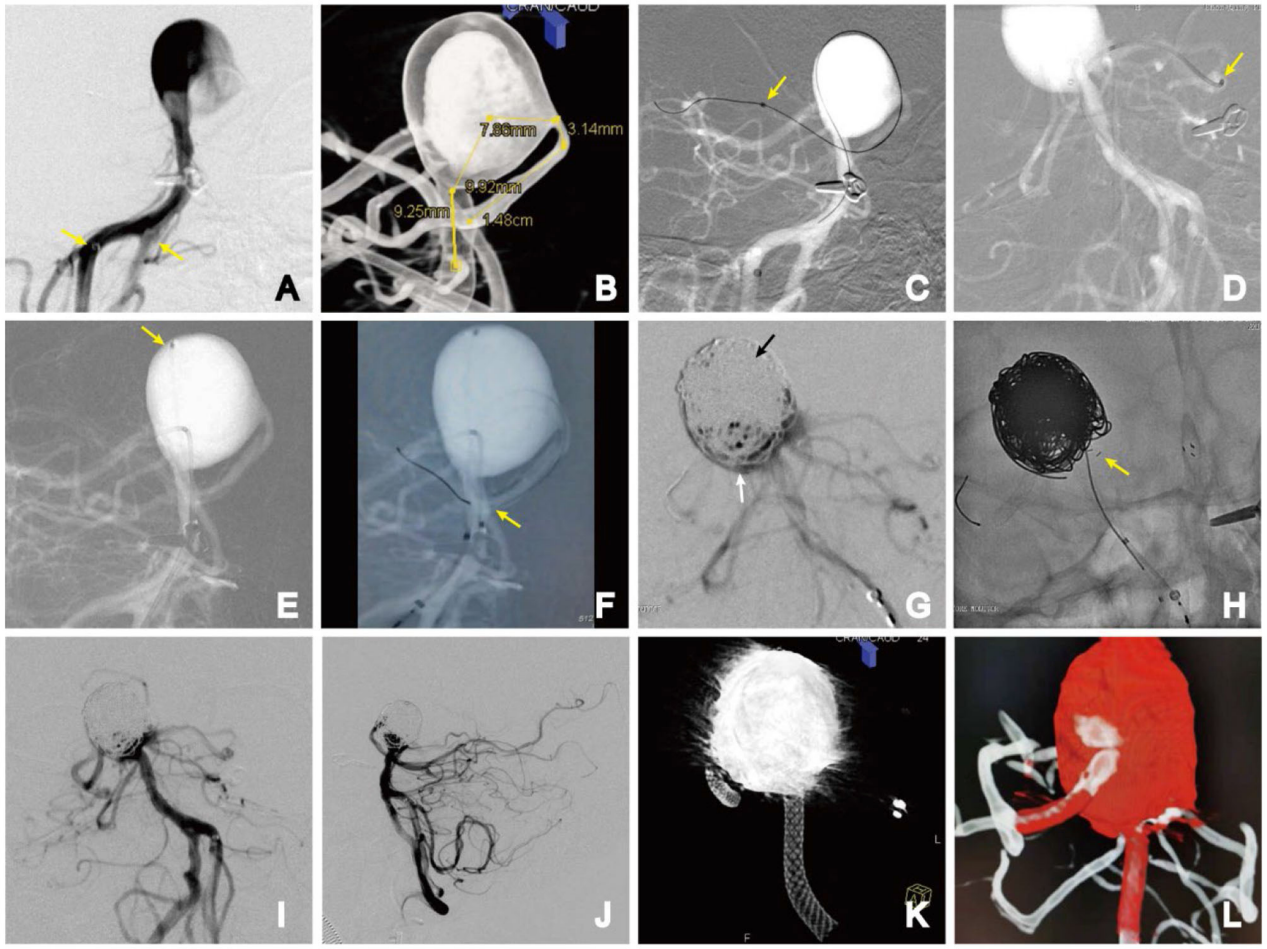
Stage 1. **(A)** Bilateral vertebral artery access (yellow arrows); **(B)** parent artery length, proximal and distal diameter, and aneurysm neck width were measured; **(C)** microguidewire-assisted microcatheter looping technique for super-select right posterior cerebral artery. The distal end of the Phenom catheter (yellow arrow); **(D)** distal end of the stent catheter in the left posterior cerebral artery (yellow arrow); **(E)** position of the coiled catheter (yellow arrow); **(F)** PED semi-deploying in the middle segment of the basilar artery. The anchor point of the distal end of the PED (yellow arrow); **(G)** densely packed upper part (black arrow) and slack coiled lower part (white arrow) of the aneurysm; **(H)** implanted E2 stent, achieving full expansion in front of the aneurysm neck in a T-fashion. AP **(I)** and lateral **(J)** DSA images; **(K)** Dyna-CT volume imaging showing the PED was fully deployed; **(L)** dual-volume reconstruction imaging showing all the vessels were patent.

The patient was put under general anesthesia, and bilateral femoral artery punctures were performed using the Seldinger method. A 5-Fr Navien catheter (Medtronic, Irvine, CA, USA) was placed in segment V3 of the left vertebral artery via a 6-Fr long sheath (Cook Medical, Indianapolis, IN, USA), while an ENVOY 6-Fr distal access guiding catheter (Codman Neuro, Raynham, MA, USA) was inserted into segment V4 of the right vertebral artery (Figure 3A). Heparin was administered intravenously based on the patient's weight to achieve a therapeutic activated coagulation time of 2–3 times the baseline, with the maintenance of a tirofiban infusion during surgery. We used 5-s digital subtraction angiography to select multiple working angles that show the distal and proximal ends of the aneurysm parent artery and to choose the appropriate working projection accordingly. The first working position was selected, and the parent artery length, proximal and distal diameter, and aneurysm neck width were measured (Figure 3B). We used a microcatheter (Synchro-14; Boston Scientific, Natick, MA, USA) assisted by a looping technique with an Echelon-10 microcatheter (Medtronic, Irvine, CA, USA) to achieve superselectivity of the right posterior cerebral artery. The Echelon-10 catheter support force facilitated the exchange of a Phenom 27 microcatheter (Medtronic) to reach the distal end of the right PCA (Figure 3C). At the second operating angle, the Prowler Select Plus microcatheter (Cordis Corporation, Bridgewater, NJ, USA) was successfully selected for the distal left posterior cerebral artery, and the Echelon-10 microcatheter was introduced and positioned in the dome of the aneurysm (Figures 3D, E).

Using a Phenom 27 microcatheter, a 4.0 × 35-mm PED was slowly advanced into the distal portion of the right posterior cerebral artery, anchored in place, and then gently semi-deployed under fluoroscopic guidance to the middle segment of the basilar artery (Figure 3F).

The coil was carefully embolized within the BAA. The middle and upper parts of the aneurysm were densely packed, while the lower part near the branch was loosely packed (Figure 3G). To avoid the coils entering the branch vessels, a 4.0 × 23-mm Enterprise-2 stent (E2, Codman, Chaska, MN, USA) was selected, which was deployed into the left PCA. The E2 tail end was placed in a T shape close to the side of the PED so that the E2 stent and PED were almost in contact without a direct metal intersection. Repeated angiography confirmed no direct blood flow, and the bilateral PCA and superior cerebellar artery (SCA) were patent. The Echelon-10 microcatheter was slowly removed, and the PED was thoroughly deployed (Figure 3H).

A control angiogram showed contrast stasis in the aneurysm dome but preserved flow through the perforator. The patient had an uneventful procedure, and recovery and remained neurologically intact (Figures 3I–L). Dual antiplatelet therapy was continued after treatment.

**Figure 4:**
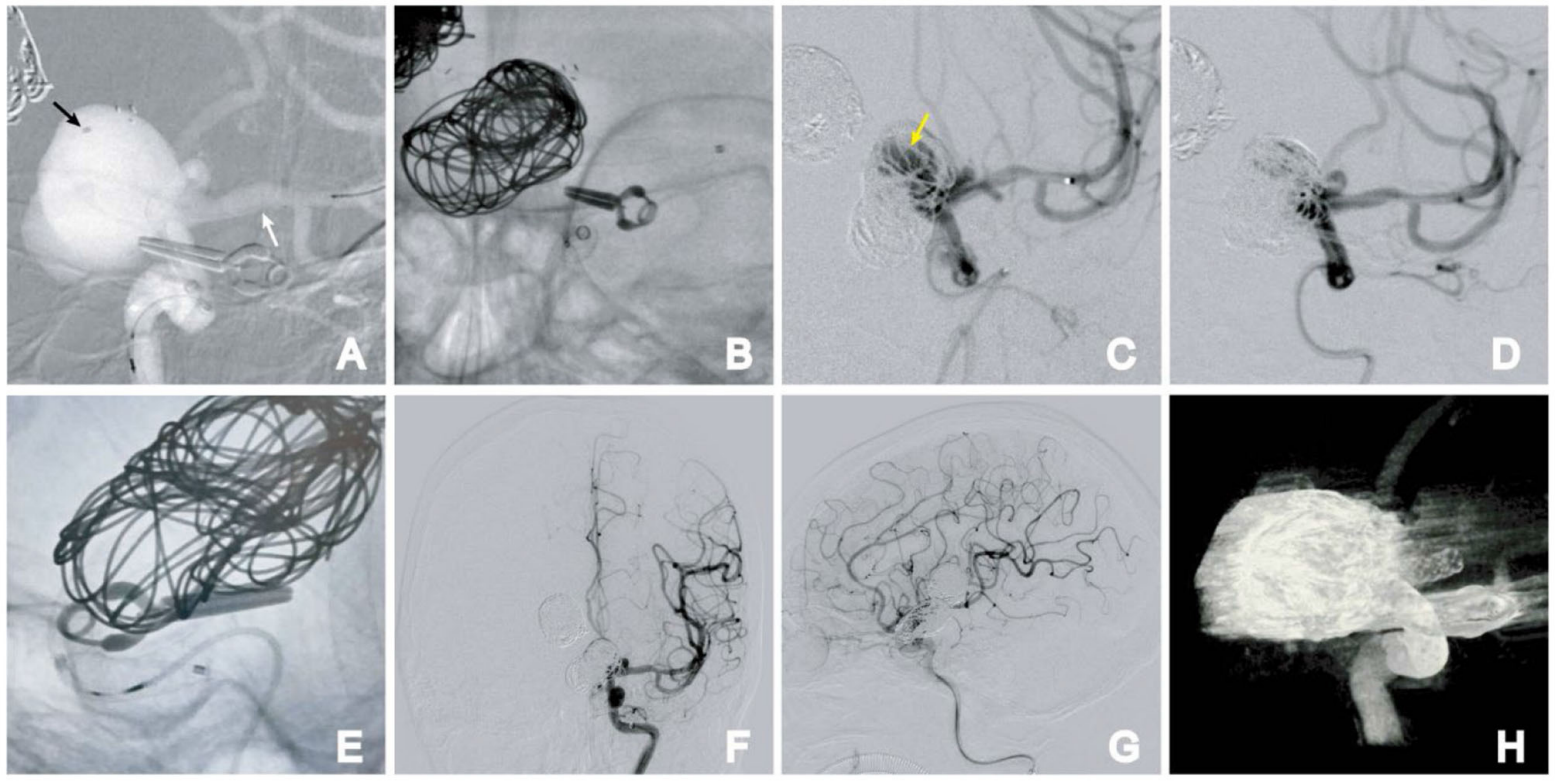
Stage 2. **(A)** Semi-deploying PED; PED anchor point (white arrow); coil catheter position (black arrow); **(B)** large-sized coil embolization was performed, followed by the complete deployment of the PED; **(C)** angiographic images reveal direct blood flow (arrow); **(D)** direct blood flow disappeared after further embolization of the aneurysm; **(E)** PED was fully deployed. **(F, G)** Postoperative frontal **(F)** and lateral **(G)** DSA images. **(H)** Dyna-CT showing the PED was fully deployed.

The patient underwent treatment for the supraclinoid aneurysm by PED-assisted coil embolization 14 days later. The procedure and postoperative course were uneventful. The PED with satisfactory apposition to the vessel wall. A control angiogram showed contrast stasis within the aneurysm dome with preserved flow through the perforator, and the patient remained neurologically intact. The patient was given dual antiplatelet therapy for 6 months after the treatment and aspirin therapy for life (Figure 4).

## 3 Results

**Figure 5:**
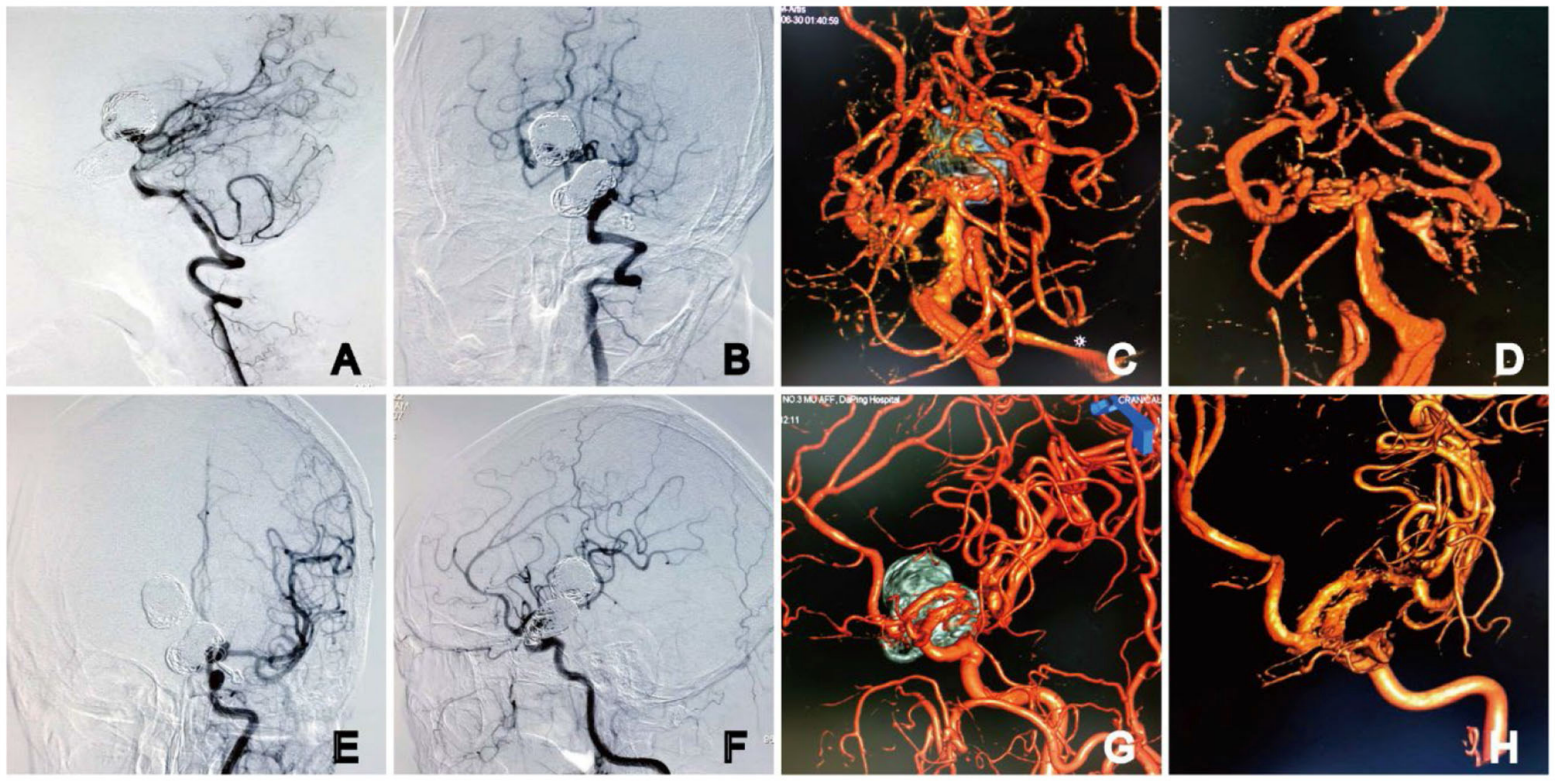
DSA at 9 months after embolization. Angiography showed complete occlusion of the basilar apex aneurysms **(A, B)** with the preservation of four channels on 3D DSA **(C, D)**. The parent artery was unobstructed in the **(E)** anteroposterior and **(F)** lateral angiography images of the left ICA. No stenosis or residual aneurysm neck was observed on 3D DSA **(G, H)**. Dual-volume **(C, G)** and single-volume reconstruction imaging **(D, H)**.

At the 9-month follow-up, the patient was asymptomatic and remained neurologically intact, with a Modified Rankin Scale (MRS) score of 0. DSA follow-ups showed complete occlusion of the aneurysm, with Raymond–Roy Occlusion Classification I (Figure 4). The patient has provided informed consent for the publication of the case. The protocol was approved by the Ethics Committee of the Daping Hospital, Army Medical University (Figure 5).

## 4 Discussion

Large and complex wide-necked basilar apex aneurysms involving many important branches, especially the posterior cerebral artery, originating from the distal end of the aneurysms, present significant technical challenges requiring different treatment approaches (1, 9). Stent-assisted coiling treatment for common basilar apex aneurysms involves the deployment of a single stent in the posterior cerebral artery, with the proximal end positioned in the basilar artery. This effectively confines the aneurysms and safeguards the contralateral posterior cerebral artery. However, this technique only partially protects against coil herniation into the parent vessel and is unsuitable for treating larger aneurysms (10, 11). While various flow diverters, such as pipeline, silk, Surpass, Tubridge, PulseRider, WEB stents, and other new materials, are available, they are not viable remedies for giant aneurysms, and employing them off-label presents certain issues (12–16). To maintain blood flow in the branch artery, traditional Y- or T-stent-assisted coil embolization has to be used to loosely fill the aneurysm cavity, resulting in incomplete aneurysm embolization and recurrence (17, 18). Moreover, the characteristics of Y-shaped stents make it challenging to reintervene recurrent aneurysms. The significance of Y-shaped stent-assisted coil embolization of the BAA is not to make the embolization denser and more complete but to support and protect the branch and blood flow reconstruction and avoid coil protrusion into the branch, leading to severe top of basilar artery syndrome (TOBS) (19).

In the past few years, the off-label use of flow diversion in basilar apex aneurysms has been attempted. More delayed aneurysm bleeding complications have been reported in the literature because the jet sign was not resolved (20). Therefore, we adopted the pipeline method combined with relatively dense coil packing. Because the contralateral PCA emanates from the aneurysms, a single-flow diversion is insufficient to reconstruct the PCA and SCA. This may lead to the escape of the coil to the PCA or SCA after embolization. Thus, if another PED is deployed in the left PCA, the diameter of the pathway needs to be enlarged, which is bound to affect blood flow in the posterior circulation and increase the risk of ischemia. At the same time, the pipeline is a braided stent that is difficult to locate and operate. Once it is not fully connected, it loses its role in blood flow reconstruction and increases the risk of perforator occlusion.

To solve the problem of the perforator on the other side, placing a different stent on the lateral side of the PED and keeping it almost in contact without direct metal intersection may allow for the reconstruction of the basilar artery apex. Therefore, we designed a “pipeline embolization device combining flow-T stenting” to reconstruct blood flow. This asymmetric stent deployment could alter the flow, favoring the PCA in which the distal end of the stent was deployed. The Enterprise-2 (E2) stent has better compliance and operability, and the supporting microguide tube is relatively thin. Hence, the E2 stent is easier to place and deploy, and the end of the E2 stent can be deployed in parallel or by “kissing”. The E2 stent has a flexible closed-cell design that allows it to be retrieved and repositioned when up to 70% of the system is deployed (21). Finally, we decided to use PED combined with E2 stent-assisted coiling.

A technical caveat to achieving this configuration involves deploying a more challenging stent first. We measured the distal diameter of the PCA at 2.2 mm and the basilar artery at 3.0 mm. The length from the distal anchor to the proximal landing was measured to be approximately 40 mm, and 4.0 × 35 mm was selected through the segmented calculation. At the same time, it was also considered that choosing a stent with a larger diameter could reduce the metal coverage rate, thereby reducing the risk of perforator occlusion. In this manner, all major parent vessels were successfully reconstructed, and the entire aneurysm neck was protected from coil herniation.

In addition, to reduce the incidence of delayed thromboembolism and long-term stenosis within the stent, the patient continued dual antiplatelet therapy for 6 months and lifelong aspirin therapy.

## 5 Conclusion

Any technology has its limitations, and although this individual case currently has no postoperative complications, longer-term clinical follow-up is still necessary. The technological and clinical outcomes attained so far are very encouraging, and we feel the pipeline embolization device combined with the flow-T stenting technique may significantly contribute to the endovascular treatment of intracranial aneurysms. This article offers treatment options as references for similar diseases. We hope to develop new types of interventional materials or a simpler way for the treatment of complex basal aneurysms.

## Data availability statement

The original contributions presented in the study are included in the article/supplementary material, further inquiries can be directed to the corresponding author.

## Ethics statement

Written informed consent was obtained from the individual(s), and minor(s)' legal guardian/next of kin, for the publication of any potentially identifiable images or data included in this article.

## Author contributions

HW and DY: conceptualization. TG: formal analysis. HY: investigation. SZ and HY: methodology. HW and LX: writing—original draft. DY: writing—review and editing. JZ: project administration, writing—original draft.
